# Artificial Neural Networks to Optimize Oil-in-Water Emulsion Stability with Orange By-Products

**DOI:** 10.3390/foods11233750

**Published:** 2022-11-22

**Authors:** Mónica Umaña, Laura Llull, José Bon, Valeria Soledad Eim, Susana Simal

**Affiliations:** 1 Department of Chemistry, University of the Balearic Islands, Ctra. Valldemossa, km. 7.5, 07122 Palma de Mallorca, Spain; 2 Analysis and Simulation of Agro-Food Processes Group, Food Technology Department, Universitat Politècnica de València, Camino de Vera s/n, 46022 Valencia, Spain

**Keywords:** orange by-products, pectins, emulsions, artificial neural network, optimization

## Abstract

The use of artificial neural networks (ANNs) is proposed to optimize the formulation of stable oil-in-water emulsions (oil 6% *w*/*w*) with a flour made from orange by-products (OBF), rich in pectins (21 g/100 g fresh matter), in different concentrations (0.95, 2.38, and 3.40% *w*/*w*), combined with or without soy proteins (0.3 and 0.6% *w*/*w*). Emulsions containing OBF were stable against coalescence and flocculation (with 2.4 and 3.4% OBF) and creaming (3.4% OBF) for 24 h; the droplets’ diameter decreased up to 44% and the viscosity increased up to 37% with higher concentrations of OBF. With the protein addition, the droplets’ diameter decreased by up to 70%, and flocculation increased. Compared with emulsions produced with purified citrus pectins (0.2 and 0.5% *w*/*w*), OBF emulsions exhibited up to 32% lower viscosities, 129% larger droplets, and 45% smaller Z potential values. Optimization solved with ANNs minimizing the droplet size and the emulsion instability resulted in OBF and protein concentrations of 3.16 and 0.14%, respectively. The experimental characteristics of the optimum emulsion closely matched those predicted by ANNs demonstrating the usefulness of the proposed method.

## 1. Introduction

Oranges are among the most cultivated and consumed fruits in the world [1]. According to the Food and Agriculture Organization, about 75 million tons of oranges was produced globally in 2020 [2]. The industrial processing of this fruit generates a considerable number of residues, including the peel, seeds, and pulp, accounting for about 55–60% of the raw fruit weight. This waste causes serious environmental pollution since it is highly organic and biologically unstable [1,3]. Nevertheless, the residues are rich in high-value compounds, including polyphenols, essential oils, carotenoids, and dietary fiber [3]. Moreover, citrus is one of the main sources of pectins, a term describing a family of anionic oligosaccharides and polysaccharides that are rich in galacturonic acid [4]. Pectins are very valuable in the food industry since they are used as gelling agents and stabilizers in confectionary, compotes, fruit juices, dairy, and so on [5]. In addition, pectins have several healthy properties, such as prebiotic potential, the capacity to reduce cholesterol plasma, the ability to protect against cardiovascular disease, etc. [5,6,7]. For these reasons, the pectin market has greatly grown in the last decade [5]. However, after the extraction of pectins, there is a large amount of discarded citrus material, and several components are still wasted [8], i.e., vitamin C and phenolic compounds, which have antioxidant activity [9]. 

Recently, pectins have also been investigated to stabilize oil-in-water emulsions [10,11,12,13]. Emulsions are thermodynamically unstable systems that consist of two immiscible liquids, one dispersed as droplets over the other. Oils and other hydrophobic compounds are incorporated/emulsified into many foods, and several food products partially or wholly consist of emulsions [14]. Furthermore, the production of an oil-in-water emulsion is generally the first step of other operations, such as lipid microencapsulation by spray drying [15]. The main objective of microencapsulation is to provide a physical barrier to an active ingredient in order to protect it from stressors that might cause oxidation or other degradation processes. Emulsions prepared for this purpose should possess certain characteristics, such as having an appropriate droplet size (droplets diameter < 10 μm), containing sufficient wall material (30–60% *w*/*w*), and still presenting a viscosity low enough to be pumped by the spray drying equipment [16]. The addition of antioxidants to an oil-in-water emulsion is an effective strategy to preserve lipids prone to oxidation [17].

The use of pectins to prepare oil-in-water emulsions is often combined with the addition of proteins [18]. Proteins are surface-active molecules that can adsorb to the surfaces of the oil droplets, lower the interfacial tension, and retard droplet coalescence [19]. Pectins, on the other hand, have been proven to increase the viscosity of the continuous phase of emulsions, hindering droplet mobility. An electrostatic interaction between pectins and proteins occurs at a pH between the isoelectric point of proteins and the pKa of the polysaccharide; thus, proteins have a positive charge, and the polysaccharide a negative one [20]. Proteins might be adsorbed in the oil/water interface, and thereafter a second layer could be constructed with the addition of negatively charged polysaccharides producing a protein–polysaccharide complex. Double-layer emulsions have been found to present better stability since the complexes of protein–polysaccharide can form a dense interfacial membrane at the water–oil interface, promoting steric repulsions [21] and also making the microencapsulation process more efficient [22]. 

Both proteins and polysaccharides coming from plants have gained attention because of the current trend of a preference for natural ingredients which do not compromise human health and/or animal welfare [23,24]. Moreover, the use of heterogeneous natural materials has the advantage of providing not only polysaccharides and/or proteins but also other interesting molecules, such as phenolic acids with antioxidant capacity. In fact, non-purified citrus materials have also aroused interest in the stabilization of emulsions [8,25]; promising results are being reported, indicating that citrus fiber promotes a Pickering effect and the formation of a fiber-based network, resulting in stable systems. 

The composition of emulsions stabilized with a protein–polysaccharide complex should be investigated and optimized, since an excess of polymers might cause segregation [20], but a very low quantity of these molecules would not be sufficient to stabilize the emulsion. Mathematical modeling is a useful and powerful tool to evaluate and optimize the formulation of food products. For instance, the composition of different types of emulsions has been previously optimized through central composite designs and response surface methodology [26,27,28,29], usually obtaining satisfactory results. On the other hand, models based on artificial neural networks (ANNs), which imitate the natural neural system, present several advantages, including high adaptability to non-linear problems, the capability to analyze complex data and find the governing rules between the corresponding factors being easy to use, and the ability to provide high accuracy in data prediction [30]. ANNs comprise a huge number of elements known as neurons, which are interconnected to form a network that can be adapted to compute a specific function [31]. The network can handle multiple independent (inputs) and dependent variables (outputs) simultaneously, thus making it useful for the optimization of food product formulation [32] or the prediction of their properties [33]. It can also optimize food industry processes [34] which have several control parameters, such as drying [35], enzymatic inactivation [36], extraction process [37], and so forth. On a few occasions, ANNs have been used to optimize the composition or the process of production of emulsions [38], mainly for pharmaceutical or cosmetic purposes [39,40].

To the best of our knowledge, no research has yet been carried out by comparing the emulsifying capacity of pure pectins with that of a heterogeneous citrus material (which naturally contains pectins) coming from a by-product. Moreover, the composition of emulsions stabilized with natural materials has not been optimized. For this reason, this study aimed to (1) evaluate the usefulness of an orange by-product flour (OBF) in the stabilization of oil-in-water emulsions and compare it with purified citrus pectins; and (2) optimize the formulation of emulsion stabilized with OBF and vegetable proteins, using ANNs application.

## 2. Materials and Methods

### 2.1. Chemical Reagents

Monohydrate citric acid, ethanol (96% and absolute), acetone, chlorohydric acid (37%), sodium hydroxide (pellets), methanol (analytic grade), ammoniac (analytic grade), Folin–Ciocâlteu reagent, and monohydrate gallic acid were purchased from Scharlau (Barcelona, Spain). The 1-methylimidazole was obtained from Merck (Barcelona, Spain). Glacial acetic acid and sorbitan monolaurate (Tween^®^ 20) were obtained from Panreac AppliChem (Barcelona, Spain). Neocuprine, 2,2′-azino-bis(3-ethylbenzo-thiazoline-6-sulphonic acid) and potassium bromide were purchased from Sigma-Aldrich (Madrid, Spain).

### 2.2. Materials

The commercial pectin (CP) (≥74% of galacturonic acid dry matter (dm)) was purchased from Sigma-Aldrich (Madrid, Spain). Glucidex^®^ maltodextrin DE 12 (MD) (Roquette, Beinheim, France) was used. The soy protein was obtained from Manufacturas Ceylan, S.L (Valencia, Spain), and the sunflower oil was obtained from a local store (Mallorca, Spain). The orange by-product flour was obtained from oranges of the Navelina variety that were purchased in a local market (Mallorca, Spain). After extracting the juice, the remaining material (by-product), composed of peel and pulp, was scalded to inactivate the endogenous enzymes, freeze-dried (LyoQuest, Telstar, Barcelona, Spain) operating at −50 °C; and vacuum pressure of 30 Pa; ground (ZM 200, Retsch^®^, Haan, Germany); and sieved (to a particle size of <0.5 mm) (FIT–0200, Filtra, Barcelona, Spain). The powder obtained is here called orange by-product flour (OBF). 

#### OBF Characteristics

The moisture content of OBF was determined according to the AOAC method no. 934.06 [41]. Protein, lipid, and ash content were determined by following the methods described by Umaña et al. [42]. The total carbohydrate content was calculated as the difference from 100 of the sum of the protein, lipids, and ash contents. The dietary fiber content was quantified with the enzymatic assay kit K-TDFR from Megazyme (Wicklow, Ireland). To characterize the fiber of OBF, its alcohol-insoluble residues were obtained as described by Eim et al. [43]. The uronic acids were quantified by colorimetry as total UA from samples hydrolyzed for 1 h at 100 °C [44]. The neutral sugars were quantified by releasing them by acid hydrolysis (Saeman hydrolysis). The released monosaccharides were converted into their alditol acetates and separated by gas–liquid chromatography, as described by Dalmau et al. [45]. The degree of methylation of pectins present in OBF and CP was determined by Fourier-transform infrared (FTIR), as described by Umaña et al. [46]. Briefly, to obtain the FTIR spectra, the samples were mixed with KBr and pressed into a pellet. The degree of methylation was determined with Equation (1) [47]:

(1) DM=124.7R+2.2013

where R is the ratio between the absorbance intensity of the band corresponding to methyl esterified carboxyl group (1740 cm^−1^) over the sum of this and the absorbance intensity of the band corresponding to the non-methyl esterified carboxyl group (1630 cm^−1^). These absorbance intensities were obtained from the FTIR spectra of the alcohol-insoluble residues of OBF (to discard molecules not corresponding to polysaccharides) and from CP. 

The antioxidant activity was determined in both OBF and CP to compare these materials. The samples were submitted to an extraction process in methanol, as described by Vallespir et al. [48], and the total phenolic compounds content was determined by using the Folin–Ciocâlteu assay. The results were expressed as mg gallic acid equivalent (GAE)/100 g dm. The antioxidant activity was measured by using the CUPRAC assay [49], and the results were expressed as mg Trolox equivalent (TE)/100 g dm. 

### 2.3. Preparation of Emulsions

The emulsions were prepared according to the compositions shown in Table 1. These compositions were chosen to evaluate the effect of (a) different concentrations of CP (0.2 and 0.5% *w*/*w*), (b) different concentrations of proteins (0, 0.3, and 0.6% *w*/*w*), and different concentrations of OBF as a substitute for commercial pectin. The quantities of CP and proteins were established according to the results of previous experiments (data not shown) and the ranges observed in the bibliography [50,51]. The OBF was evaluated as a substitute for the commercial pectins; thus, the amount of pectins present in the OBF was estimated from the uronic acids and neutral sugars analysis, considering that pectins are generally composed of uronic acids, arabinose, galactose, and rhamnose [4]. Thereafter, the amount of OBF added to the emulsions was equivalent to the concentrations of pectins investigated (e.g., 0.2% *w*/*w* of CP is equivalent to 0.95% *w*/*w* of OBF), and an extra concentration of OBF (3.4% *w*/*w*) was prepared to evaluate the possibility of working with higher amounts of this material since it could provide interesting compounds, such as antioxidants and fiber. A control emulsion was obtained without any emulsifier. All the emulsions contained 6% *w*/*w* of oil and 40% *w*/*w* of dry matter. MD was used as wall material for the preparation of emulsion with a typical composition for further spray drying. The MD content was adjusted to maintain the same proportion of dry matter in all the emulsions. 

As can be seen in Table 1, emulsions were classified into 6 batches: control (no emulsifiers), PR (single-layer emulsions containing only proteins), CP (containing only commercial pectins), CP-PR (double-layer emulsions containing both protein and commercial pectins), OBF (containing only the OBF), and OBF-PR (double-layer emulsions containing both the OBF and proteins). Emulsions were named according to their composition; thus, the concentration of pectin is indicated as 0.2CP and 0.5CP (for those emulsions containing purified commercial pectin) or 1.0OBF, 2.4OBF, and 3.4OBF for those containing the orange by-product flour. Thereafter, if the emulsion contained protein, its concentration is indicated as 0.3PR or 0.6PR. Every emulsion (250 g) was prepared as described below, at least in duplicate.

A sodium acetate buffer (1.2 M, pH 3.5) was prepared. This pH (3.5) was chosen since it is below the isoelectric point of soy proteins, and a positive charge of this molecule should be expected under these conditions [52]. 

The control emulsion was prepared without any emulsifier, according to the protocol described by Hernández et al. [16], with some modifications. Briefly, an initial oil-in-water emulsion was prepared with about 15% of the water needed in the formulation as acetate buffer, using an Ultra-Turrax© (T25 Digital, IKA, Königswinter, Germany) at 16,000 rpm for 10 min. Then it was diluted with an aqueous solution of MD, which contained the rest of the buffer (85%), and submitted to 5 min of agitation with the Ultra-Turrax© at 16,000 rpm. 

In the case of the PR batch, (single layer, 0.3PR and 0.6PR), half of the water of the formulation was used as acetate buffer to dissolve MD. Proteins are not very soluble at pH 3.5; thus, to hydrate them, they were added to the rest of the water, magnetically stirred for 2 h, and hydrated overnight [53]. Thereafter, the pH of the protein suspension was adjusted to 3.5 with HCl (4.8 M). The emulsion was prepared by adding the oil to the protein suspension and homogenizing it with an Ultra-Turrax© for 10 min at 16,000 rpm. Finally, the emulsion prepared with the protein suspension was diluted with the MD solution, homogenizing it with an Ultra-Turrax© (16,000 rpm) for 5 min more.

For the CP batch, the CP was dissolved in acetate buffer by magnetically stirring for 2 h and then hydrated overnight. Then the emulsion was directly produced by adding the oil and homogenizing it for 15 min in the Ultra-Turrax© at 16,000 rpm.

For the CP–PR batch, the emulsions were prepared by using the layer-by-layer technique described by Moser et al. [18], with some modifications. Proteins and CP were hydrated as described for the PR and CP batches, respectively; half of the water of the formulation was used for proteins, and the rest was used as acetate buffer for CP. The MD was dissolved in the CP solution. The emulsion was prepared by adding the oil to the protein suspension (previously adjusted to a pH 3.5), as described for the PR batch. The emulsion was diluted with the solution of CP and MD homogenizing for 5 min (with Ultra-Turrax© at 16,000 rpm).

In the case of the OBF batch, emulsions containing only OBF, the MD was previously dissolved in the buffer acetate. Thereafter, the OBF was added and homogenized for 8 min with the Ultra-Turrax© at 16,000 rpm to reduce the particle size of the OBF. The emulsion was directly produced by adding the oil as described for the CP batch.

Finally, for the OBF-PR batch, the layer-by-layer technique described for the CP–PR batch was used. Proteins were hydrated in half of the water of the formulation, as described for the PR batches. The MD was dissolved in buffer acetate, and the OBF was added and homogenized as explained for the OBF batch. The emulsion was prepared by adding the oil to the protein suspension (previously adjusted to a pH 3.5), as described for the PR batch. The emulsion was diluted with the mix of OBF and MD, homogenizing for 5 min (with Ultra-Turrax© at 16,000 rpm).

All the homogenization steps were carried out in an ice-water bath, and the temperature of the sample was controlled and maintained at below 40 °C during the process. Overall, all the emulsions were produced by homogenizing with the oil for a total time of 15 min. 

### 2.4. Emulsions’ Characteristics

#### 2.4.1. Apparent Viscosity

The apparent viscosity of the emulsions was determined with a rotational viscometer (ST-DIGIT R, J.P. Selecta^®^, Barcelona, Spain) at 25 °C, using a spindle of 47 mm and 35 mm for more viscous emulsions with a velocity of 200 rpm (velocity gradient γ (s^−1^) of 53 s^−1^).

#### 2.4.2. Z Potential

The Z potential was measured by using a Nano Zetasizer (Nano ZS90, Malvern, UK). First, the emulsions were diluted (1/500) to avoid multiple scattering effects [54]. The pH of this dilution was adjusted to the original pH (3.5). The Z potential was measured at 25 °C and determined with the Smoluchowsky model by measuring the electrophoretic mobility of the droplets.

#### 2.4.3. Droplet Size Distribution

The droplet size distribution of the emulsions was determined with image analysis of photographs taken with an optical microscope (BH2, Olympus, Barcelona, Spain) connected to a digital camera (C-B10+, Optika, Bergamo, Italy). The emulsions were diluted with acetate buffer (3.5) (3:20 *v*/*v* emulsion:buffer) and immediately observed with the microscope, using a 20× objective. Micrographs (at least 10 per sample) were analyzed with Fiji software [55,56]. The micrograph contrast was enhanced (5% normalized), and micrographs were transformed into binary. Thereafter, the Gaussian Blur filter was applied (sigma = 2), followed by the “Classic Watershed” segmentation option within the “MorphoLibj” library [55] to construct a boundary for each droplet. The area of the droplets was determined automatically, using a scale with a known standard. Using the area and the assumption that the particles were spherical, the diameter and volume of each droplet were estimated. The results were expressed as % volume distribution vs. particle size [57]. The percentiles 10, 50, and 90 (d10, d50, and d90) were obtained from the droplet size distribution. 

#### 2.4.4. Flocculation

The percentage of flocculation of the emulsions was also measured by using image analysis with Fiji software [55,56]. After the processing described in the previous section, Section 2.4.1, the micrographs were submitted for further analysis by applying the “Dilate” command to convert each floc into a whole particle. Thereafter, the program was settled to analyze particles excluding those with a size below the area of the largest particle measured in the previous analysis; thus, all particles not belonging to floccules were eliminated. The images obtained from this process were transformed by applying the “Minimum filter” (2 pixels) to reduce the size of the floccules which were previously dilated. Then the floccules were again separated into individual particles with the “Watershed” command. The area of each droplet belonging to the floccules was automatically obtained as described in Section 2.4.3, and the volume was calculated. The percentage of flocculation was expressed as the volume percentage of flocculated oil.

#### 2.4.5. Creaming Index

The creaming index of the emulsions was measured following the method described by Edris et al. [58]. The emulsions (10 mL) were poured into a 10 mL test tube and left undisturbed at room temperature (~22 °C). Using a Vernier caliper, the sample’s total height (TH) and the layer formed at the bottom of the test tube (SH) were measured after 24 h. The creaming index was calculated by using Equation (2):

(2) $$\mathrm{Creaming}\;\mathrm{index}=\frac{\mathrm{SH}}{\mathrm{TH}}\times 100$$

The lower the creaming index value, the more stable the emulsion was against droplet migration to the top.

### 2.5. Statistical Analysis

All determinations were carried out at least in duplicate, and the results were presented as average ± deviation standard. To evaluate if there were significant (*p* < 0.05) differences among the emulsions with different compositions, the parametric test ANOVA was applied, and thereafter means were compared with Tukey’s [59] test, using RStudio 2 February 2022 [60]. The percentiles and the level of flocculation of the emulsions measured immediately after their preparation and after 24 h were compared with Student’s *t*-test (*p* < 0.05). 

### 2.6. Emulsion Optimization Using ANNs

Before optimizing the formulation to obtain stable oil-in-water emulsions containing the OBF and with adequate technological properties, the emulsion characteristics were modeled by using ANNs. A 2-way ANOVA was applied, considering the OBF and protein content as factors to evaluate if they significantly (*p* < 0.05) affected the characteristics of the emulsions.

#### 2.6.1. Modeling with Artificial Neural Networks

The artificial neural networks (ANNs) were developed in order to model the influence of both the OBF content (from 0 to 3.40%) and protein content (from 0 to 0.60%) (inputs) on the emulsion’s characteristics (apparent viscosity; d10, d50, and d90 percentiles; Z potential; percentage of flocculation; and creaming index) (outputs). 

The design of the ANNs considered the most common architecture, based on a multilayer feed-forward structure and computing the ANN weights and biases with the back-propagation training algorithm. For figuring out the necessary hidden layers and nodes, there is no set rule. However, the use of two or more hidden layers has only sometimes proven to be advantageous, with one hidden layer typically being sufficient in most situations. The number of outputs of the experimental data was relatively high; thus, a neural network was developed for each variable output with one hidden layer. In this way, we ensured that the number of parameters of the neural network was considerably smaller than the number of experimental data. The number of neurons (between 2 and 5) and the transfer functions (tansig, logsig, and purelin) in the hidden and output layer were examined. 

The Deep Learning Toolbox of Matlab^®^ R2022a was used (The Mathworks, Natick, MA, USA, 2022) to develop each ANN. The “fitnet” function was applied to develop a feed-forward backpropagation network, and with the “train” function, the ANNs were trained to establish the number of neurons in the hidden layer. To reduce mean square errors (MSEs) between the network’s outputs and the experimental data, the parameter values were updated through a training approach. The Bayesian regularization method was used during neural network training to prevent the problem of over-fitting [61], and each ANN’s input and target vectors were partitioned at random, with 80% used for training and 20% for testing. This method updates the weight and bias values according to the Levenberg–Marquardt optimization algorithm [62,63]. It minimizes a combination of the MSEs and weights and then selects the best combination to create a network that generalizes well.

The overall (taking into account all the experimental and calculated data) correlation coefficient value, the overall MSE value, and the MSEs and the correlation coefficients estimated in each of the three stages of the ANN analysis (training and testing) were evaluated to select an adequate number of neurons to obtain the smallest number of weights and biases. Before the ANNs’ development, all the inputs and outputs’ data were normalized to give them values between −1 and 1. 

Finally, to evaluate the accuracy of the model, the mean relative error (MRE) was calculated by comparing the experimental (Z_exp_) and calculated (Z_calc_) data, using Equation (3), where N is the number of experimental data. For those characteristics presenting experimental results of 0% (creaming and flocculation), the experimental and calculated data were first transformed by subtracting from 100 the calculated or experimental value. The model was also evaluated by plotting the calculated vs. the experimental data and obtaining the correlation coefficient (r^2^), the slope, and the y-intercept of the linear regression. The prediction bounds (95%) were obtained with the “predint” function of Matlab^®^ R2022a. The residuals (differences between experimental and calculated values) were also obtained, and their normality was evaluated by plotting their histogram and carrying out the Shapiro test with RStudio 2 February 2022 [60]. The residuals were plotted versus the calculated data to evaluate if they behaved randomly.

(3) $$\mathrm{MRE}\;(\%)=\frac{100}{N}\sum_{i=1}^{n}\frac{\left|Z_{\exp}-Z_{\mathrm{calc}}\right|}{Z_{\exp}}$$

To better observe the simulation obtained with the ANNs, the “contourf” function of MATLAB^®^ R2022a (The MathWorks, 2021) was used to create filled contour plots containing isolines of each emulsion characteristic on a protein-content–OBF content plane.

#### 2.6.2. Optimization Problem Formulation

The emulsions’ characteristics that describe their stability and the important parameters for future spray drying were established as decision variables of the optimization problem. Since emulsions with droplets larger than 10 μm are undesirable because they result in poor encapsulation efficiency, d90 < 10 μm was settled as a constraint [64]. Emulsions with high viscosities are not convenient for spray-drying processes since they hinder both the pumping step [65] and the droplets’ formation during atomization [64]. Therefore, a constraint of apparent viscosity < 200 mPa·s was also established. Generally, smaller droplets without flocculation or migration indicate higher stability of the emulsion [66] and better results during microencapsulation processes [16], so those parameters need to be minimized. 

Therefore, the optimization problem consisted of finding the values of the decision variables (OBF and proteins concentration) that minimize the d50, percentage of flocculation, and creaming index by considering two constraints: d90 < 10 μm and viscosity < 200 mPa·s. To calculate the objective function and to check that the constraints were met, the previously trained ANNs were used to predict all the variables, as well as the d10 and Z potential, which were not included in the objective function. The average values of the decision variables were used as initial values for the optimization process.

To solve the optimization problem, the function “surrogateopt” of MATLAB was applied to get close to the global optimum, and subsequently, the function “fmincon” was used to obtain the optimum with higher accuracy. 

Finally, an emulsion with the obtained optimum composition was experimentally prepared and characterized by using the same analyses as for the rest of the emulsions. The experimental results of the optimum emulsion characteristics were compared with those predicted by the ANNs, considering their prediction bounds (95%), using Student’s *t*-test (*p* < 0.01 and *p* < 0.05) with RStudio 2 February 2022 [60].

## 3. Results and Discussions

### 3.1. OBF Characteristics

The composition of the OBF is shown in Table 2. It was mainly composed of carbohydrates, and small amounts of proteins, ashes, and lipids were observed. Generally, this composition is similar to that previously reported for orange by-products (pulp, peel, and mixtures of them) [3,67,68,69]. Some differences in the protein and total fiber content which, according to some studies, are in the range of 6–9 and 54–63 g/100 g dm, respectively [68,69,70], might be explained by the different varieties of oranges (a mixture of Ruby and Hamlin was used by Macagnan et al. [69]) and/or the fact that, in our study, we used both pulp and peel, and some of the other investigations used only the peel [70].

The total content of pectins was estimated from the carbohydrate composition of the fiber, considering that pectins are mainly composed of uronic acid (homogalacturonan chains) and some neutral sugars such as rhamnose, which conform to the rhamnogalacturonans I chains, along with other sugars, mainly arabinose and galactose [4]. As expected, uronic acid was the main figure of this material, indicating a high pectin content, which was very similar to that previously reported in orange by-products after juice extraction (both pulp and peel; about 25 g/100 g dm) [71,72]. The pectins present in the OBF showed a degree of methylation that would classify them as low-methoxyl pectins (degree of methylation < 50%) [73]. This parameter was also measured for CP, presenting a similar value to that observed in OBF (47.98 ± 1.92%) and would also be classified as low-methoxyl pectins. The presence of low-methoxyl pectins in emulsions stabilized by a proteins–pectin complex is desirable since it indicates a large presence of negatively charged carboxylic groups, which ensure the interaction with the positively charged proteins [12]. 

The values obtained for total polyphenols and antioxidant activity were similar to those reported by Reference [74] (about 17 mg GAE/g dm and 27 mg TE/g dm for total polyphenols and antioxidant activity according to CUPRAC, respectively) in orange by-products (pulp and peel). These determinations were also carried out in CP; the results obtained were 0.27 ± 0.01 mg GAE/g dm for total polyphenols content and 2.24 ± 0.60 mg TE/g dm of antioxidant activity according to CUPRAC. As can be seen, the total polyphenols content of OBF was about 60-fold higher than that of the purified citrus pectin (CP), and the antioxidant activity was about 10-fold higher. The process of extraction and purification of citrus pectins results in the loss of molecules such as polyphenols and probably other antioxidant compounds. 

### 3.2. Emulsion Characteristics

The emulsions prepared with OBF or CP with and without proteins were characterized in terms of apparent viscosity, Z potential, droplet size distribution, and creaming index. The control emulsion was white and more transparent than the rest. Emulsions produced with CP and/or proteins had a whitish color, and those containing OBF presented a color between yellow and orange (Supplementary Figure S1).

#### 3.2.1. Apparent Viscosity

**Figure 1:**
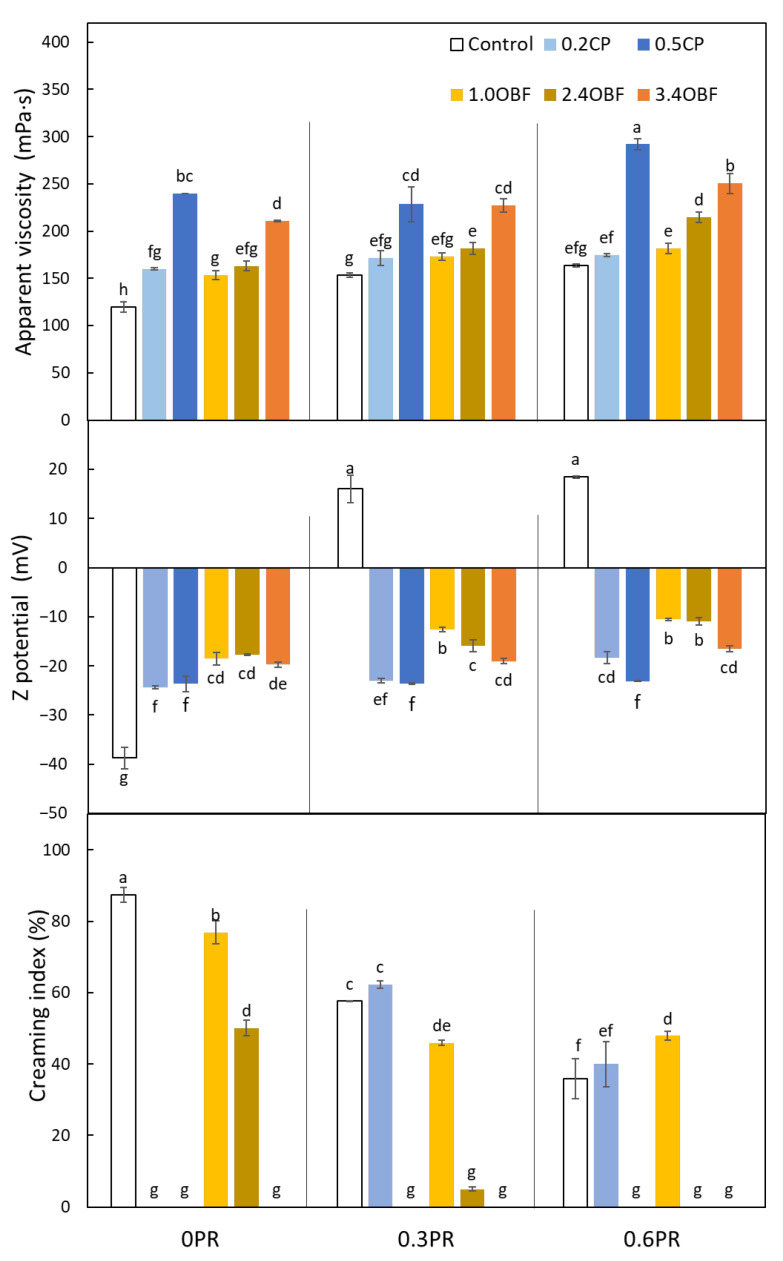
Characteristics of oil-in-water emulsions prepared without any source of pectins or proteins (control), containing purified commercial pectins (0.2CP and 0.5CP for 0.2 and 0.5% *w*/*w*, respectively) or an orange by-product flour (1.0OBF, 2.4OBF, and 3.4OBF for 0.95, 2.38, and 3.40% *w*/*w*, respectively) combined with proteins (0.3PR and 0.6PR for 0.3 and 0.6% *w*/*w*, respectively) or without them (0PR). Different letters indicate significant differences (*p* < 0.05).

The apparent viscosity of the emulsions is presented in Figure 1. As can be seen, all the emulsions containing at least one emulsifier, either CP, OBF, or/and protein, were more viscous than the control emulsion. Increasing the CP concentration resulted in higher viscosities. For instance, 0.5CP emulsion was about 100 and 50% more viscous than the control and the 0.2CP emulsions, respectively. Pectins are known for their ability to increase the viscosity of water [13]. These polysaccharides form gels through the connection of the homogalacturonan junction zones, making a network that traps water and other molecules [4]. Increasing the OBF concentration also resulted in greater viscosities, with this increase being significant (*p* < 0.05) only when comparing 1.0OBF and 2.4OBF with 3.4OBF. For instance, the 3.4OBF emulsion was about 37% more viscous than the 1.0OBF emulsions. When comparing CP and OBF in equivalent quantities of pectins (e.g., comparing 0.2CP with 1.0OBF, and 0.5CP with 2.4OBF), it can be observed that the viscosities did not present significant (*p* > 0.05) differences in the smallest quantities of pectins (0.2CP). However, the emulsion 0.5CP was significantly (*p* < 0.05) more viscous than the emulsions 2.4OBF. This indicates that, probably, not all the pectins present in OBF were completely dissolved in the continuous phase of the emulsion. 

Regarding the influence of proteins, generally, higher viscosities were observed when increasing the protein concentration. This effect was more evident when the protein was increased up to 0.6% *w*/*w*. Double-layer emulsions were significantly (*p* < 0.05) more viscous than emulsions containing only CP or OBF (single-layer) exclusively when the protein concentration was 0.6% *w*/*w*. Francisco et al. [75] also observed a small increase in the viscosity of oil-in-water emulsions when increasing the soy protein concentration. 

#### 3.2.2. Z Potential

The results corresponding to the Z potential of the emulsions are also shown in Figure 1. The 0.3PR and 0.6PR emulsions were the only ones that presented a positive value of Z potential. This confirms that the proteins were charged positively, meaning that a pH of 3.5 was below their isoelectric point. 

In the case of the control emulsion, the high negative value observed could be explained by an excess of OH^−^ ions adsorption [76]. The emulsions containing only CP showed negative values of Z potential, which was expected considering that the pectins present negatively charged carboxyl groups (COO^−^). No significant differences (*p* > 0.05) between emulsions 0.2CP and 0.5CP were observed. The emulsions containing only OBF also showed negative values, with no significant (*p* > 0.05) differences among them. The absolute values were similar but lower than those reported by Huang et al. [77] (from −30 to −20 mV), who stabilized oil-in-water (4.5% of oil *v*/*v*) emulsions with orange pulp (1.5% *w*/*v*). When comparing emulsions containing only CP or only OBF, it can be observed that the latter (OBF) have significantly (*p* < 0.05) lower absolute values of Z potential. Thus, probably not all pectins present in this material were dissolved; this coincides with the observation made for the viscosity results. In addition, it should be considered that OBF is a heterogenous material which, besides pectins, contains other molecules such as proteins or polyphenols that might affect the Z potential. The addition of proteins to the emulsions containing CP resulted in a decrease of the absolute value of this parameter only on the 0.2CP_0.6PR emulsion. However, when adding proteins to the emulsions containing OBF, the Z potential showed significant (*p* < 0.05) decreases in 1.0OBF emulsions even when adding only 0.3% *w*/*w* of proteins. Furthermore, in 2.4OBF, this decrease was only significant (*p* < 0.05) when proteins represented 0.6% *w*/*w* of the emulsion. The proteins were not observed to have any effect on the Z potential in 3.4OBF emulsions. The negative value of all emulsions containing CP or OBF with proteins indicates that pectins saturated the protein interface [78], and high contents of pectins (0.5CP and 3.4OBF) did not allow a decrease of this value when adding proteins. 

Z potential values provide some information about emulsion stability, and it is generally accepted that larger absolute values of this parameter mean greater electrostatic repulsions between the droplets [79]. According to this, emulsions 0.3PR, 0.6PR, and 1.0OBF containing both concentrations of proteins (0.3 and 0.6% *w*/*w*) and emulsion 2.4OBF_0.6PR would show poor electrostatic repulsion. Moser et al. [18] investigated emulsions prepared with chickpea proteins with or without high-methoxyl pectins and reported a low absolute value of Z potential of emulsions prepared with proteins–pectin complexes (−4.6 mV) as well. However, these types of complexes might be able to stabilize emulsions by steric rather than electrostatic repulsions [10].

#### 3.2.3. Droplet Size Distribution

**Figure 2:**
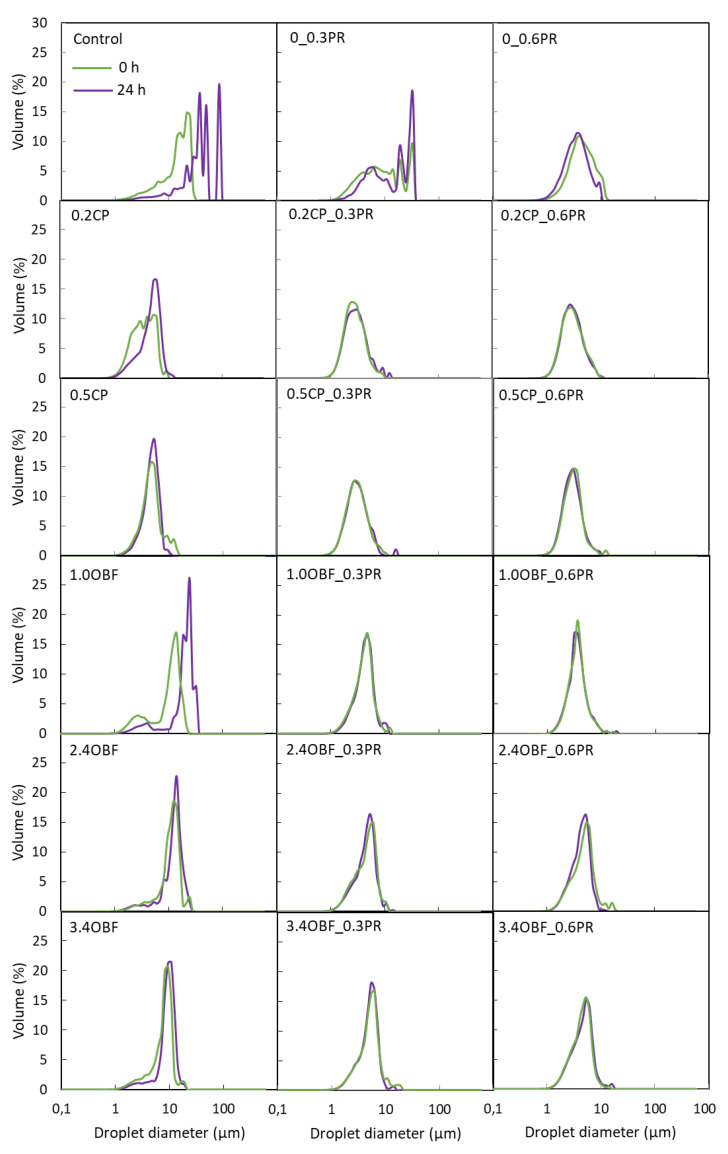
Droplet size distribution of oil-in-water emulsions obtained immediately after their preparation (0 h) and after 24 h without any source of pectins or proteins (control), containing purified commercial pectins (0.2CP and 0.5CP corresponding to 0.2 and 0.5% *w*/*w*, respectively) or an orange by-product flour (1.0OBF, 2.4OBF, and 3.4OBF corresponding to 0.95, 2.38, and 3.40% *w*/*w*, respectively) combined with proteins (0.3PR and 0.6PR corresponding to 0.3 and 0.6% *w*/*w*, respectively) or without them (0PR).

**Figure 3:**
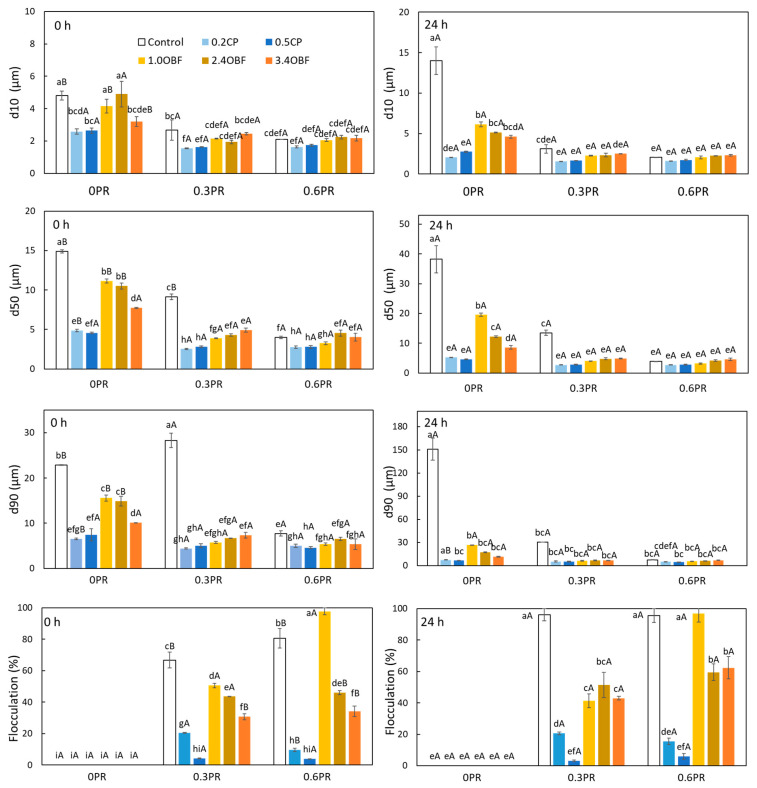
Percentiles of the droplet size distribution and percentage of flocculation of emulsions obtained immediately after their preparation (0 h, **left**) and after 24 h (**right**), without any source of pectins or proteins (control), containing purified commercial pectins (0.2CP and 0.5CP for 0.2 and 0.5% *w*/*w*, respectively) or an orange by-product flour (1.0OBF, 2.4OBF, and 3.4OBF for 0.95, 2.38, and 3.40% *w*/*w*, respectively) combined with proteins (0.3PR and 0.6PR for 0.3 and 0.6% *w*/*w*, respectively) or without them (0PR). Different lowercase letters indicate significant (*p* < 0.05) differences among samples with different composition, and different uppercase letter means significant differences (*p* < 0.05) between the same sample at 0 and 24 h.

**Figure 4:**
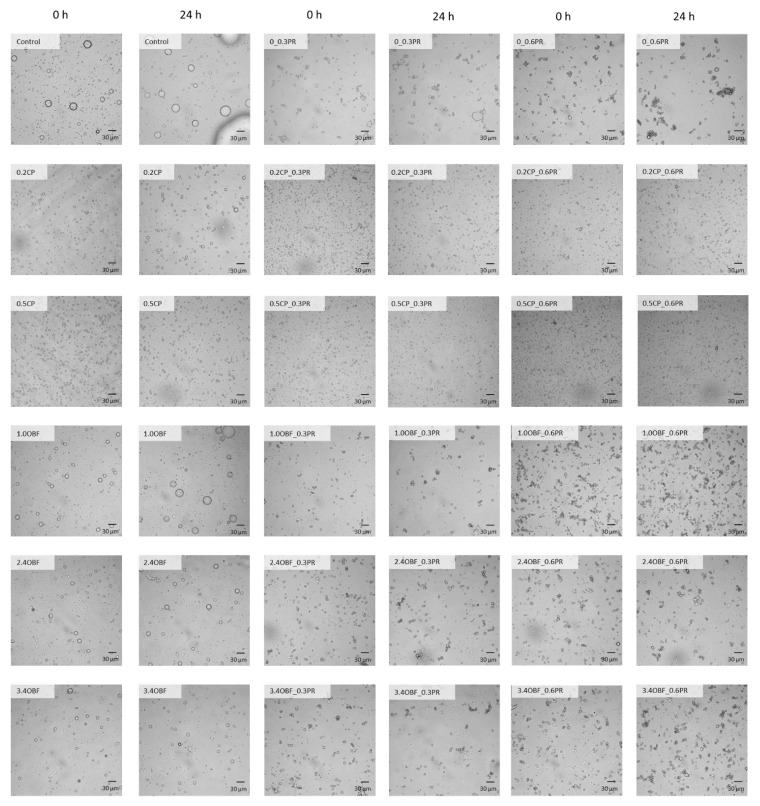
Micrographs of oil-in-water emulsions obtained with the optical microscope immediately after their preparation (0 h) or after 24 h, without any source of pectins or proteins (control), containing purified commercial pectins (0.2CP and 0.5CP for 0.2 and 0.5% *w*/*w*, respectively) or an orange by-product flour (1.0OBF, 2.4OBF, and 3.4OBF for 0.95, 2.38, and 3.40% *w*/*w*, respectively) combined with proteins (0.3PR and 0.6PR for 0.3 and 0.6% *w*/*w*, respectively) or without them (0PR).

The droplet size distributions of each emulsion obtained at 0 and 24 h are presented in Figure 2, and the percentiles d10, d50, and d90 of the distributions and the percentage of flocculation are shown in Figure 3. In addition, a micrograph of each emulsion is shown in Figure 4. As can be seen in Figure 2, all the emulsions, except for the control and 0.3PR, presented a monomodal distribution. According to the median diameter (d50) presented in Figure 3, the control emulsions and 0.3PR presented the largest droplets, the absence or the presence of a too-low amount of emulsifier resulted in emulsions with big droplets and poor stability. The addition of pectins from both CP and OBF clearly decreased the median diameter of the droplets. For instance, 0.2CP and 1.0OBF showed a median diameter (~4.9 and 11.1 μm, respectively) of about 67 and 25% smaller than the control emulsion (~14.9 μm). This effect was greater when adding CP rather than OBF. However, when increasing the OBF concentration from 0.95 to 3.4% *w*/*w*, the diameter of the droplets significantly (*p* < 0.05) decreased. The stabilization effect of polysaccharides in emulsions is usually related to their capacity to increase the viscosity of water and modify its rheological characteristics by spontaneous macromolecular gelation or structuration [80]. Moreover, the emulsifying effect of pectic polysaccharides has already been reported for purified pectins coming from different natural sources. For instance, Mendez et al. [81] observed that when increasing the concentration of pectin extracted from watermelon (from 1.5 to 3.5% *w*/*w*) in oil-in-water emulsions, the droplet size of the emulsions decreased. They explained that the side chains of neutral sugars (RG-I) of pectins macromolecules were responsible for preventing the oil droplet coalescence through steric stabilization. On the other hand, double-layer emulsions generally showed smaller droplets than the emulsions stabilized only with CP or OBF (0PR). For instance, the emulsions 0.2CP_0.3PR and 1.0OBF_0.3PR showed a d50 (~2.5 and 3.9 μm, respectively) that was about 48 and 65% smaller than that observed for those emulsions without proteins. However, the addition of proteins also promoted flocculation. This can be observed in Figure 4 and through the percentage of flocculation represented in Figure 3. Generally, the emulsions produced without proteins presented individual droplets with relatively large sizes (Figure 4) and no flocculation (Figure 3), whilst the emulsions containing proteins showed smaller clustered droplets. In fact, those emulsions prepared only with proteins were highly flocculated (about 67 and 80% of the volume of oil was flocculated in 0.3PR and 0.6PR emulsions, respectively). Soy proteins are mainly formed by globular proteins (about 70%), which are not able to avoid droplet aggregation by steric repulsion alone because they create thin interfacial layers [19]. Still, they can avoid flocculation through strong electrical charges [82], as long as the emulsions present a pH value sufficiently above or below that of the proteins’ isoelectric point. According to the results presented for the Z potential, emulsions prepared with proteins presented low absolute values of this parameter, meaning that low electrical charges were observed. Hence, the probability of flocculation was high. Moreover, food proteins are not very soluble in water, especially when they are close to their isoelectric point; thus, they can be found in the emulsion as supramolecular aggregates or solid particles. These particles or aggregates stabilize emulsions through a “Pickering” effect, but they might also act as a “bridge” between two or more droplets, causing bridging flocculation [80].

In combination with proteins, significantly (*p* < 0.05) higher percentages of flocculation were observed in emulsions containing OBF than in those produced with CP. This could be because OBF contained other molecules besides pectins which might increase the attraction among droplets, and it also contained insoluble material that might increase the Pickering effect. However, the percentage of flocculation significantly (*p* < 0.05) decreased when increasing the OBF concentration. This could be explained by the increase of viscosity when adding higher amounts of OBF or because adding more pectins improved the formation of the protein–pectin complex, increasing the possibility of steric repulsions. 

The measurement of the droplet size distribution over time provides information about the emulsion stability. It can be seen in Figure 2 that the 0, 0.2CP, and 1.0OBF emulsions shifted to the right after 24 h, indicating that larger droplets were observed. In fact, for these emulsions, the d50 increased by 157, 7.9, and 75% after 24 h of their preparation, respectively. On the other hand, the double-layer emulsions were highly stable against droplet size variation; this could be inferred from their coincident size distributions over time (Figure 2) and from their percentiles, which showed no significant (*p* > 0.05) changes after 24 h (Figure 3). Therefore, even when the protein addition increased the percentage of flocculation of the emulsions, they also prevented coalescence over time, meaning that the floccules did not evolve into larger individual droplets. Regarding the stability against flocculation, those emulsions containing only pectins (CP or OBF) kept their microstructure without flocculation over time. Meanwhile, those emulsions containing only proteins significantly (*p* < 0.05) increased their flocculation percentage after 24 h. The emulsions prepared with CP and proteins maintained their percentages of flocculation over time. The emulsion containing OBF and proteins showed different trends; on the one hand, 1.0OBF emulsions with proteins, which were already highly flocculated, maintained the same level over time, while those containing 2.4OBF with proteins increased their percentage of flocculation only when proteins represented 0.6% *w*/*w*. Finally, 3.4OBF_0.3PR and 3.4OBF_0.6PR, which initially were less flocculated, significantly (*p* < 0.05) increased their flocculation after 24 h to obtain similar percentages to those with 2.4OBF and proteins. 

#### 3.2.4. Creaming Index

The creaming index results (Figure 1) indicate that the addition of CP significantly (*p* < 0.05) reduced the droplet migration; thus, after 24 h, no creaming was observed for 0.2CP and 0.5CP emulsions. The addition of OBF also decreased the droplet migration compared with the control emulsion. Thus, for emulsions 1.0OBF and 2.4OBF, the creaming index was about 12 and 43% lower than that of the emulsion 0. Moreover, for 3.4OBF, no creaming was observed. In the same way, Qi et al. [8] reported that oil-in-water emulsions (25% *v*/*v* of oil) produced with citrus fiber were more stable against creaming when increasing the concentration of this material (from 0.25 to 3.0% *w*/*v*). These results could be explained by the increase of the viscosity observed when adding CP and OBF, larger viscosities hindering the droplets’ mobility (Stokes law) and/or by the fiber network formation that traps the droplets [8]. When comparing the emulsions with an equivalent amount of pectins from CP and OBF, larger creaming values were observed for the OBF emulsions, and this coincides with the results described for the viscosity, since they indicated that larger amounts of OBF are needed to obtain similar viscosities than those observed with CP. 

The addition of proteins as the only emulsifier significantly (*p* < 0.05) decreased the creaming index of the emulsions compared with the control emulsion. However, when proteins were added to the emulsions containing 0.2% *w*/*w* CP, the creaming index increased from 0 to about 60 and 40% for 0.3 and 0.6% *w*/*w* of proteins, respectively. This could be explained by the increase in the level of flocculation when adding proteins. The presence of floccules, in relatively dilute emulsions, results in a higher creaming index [57,80]. The addition of proteins in 0.5CP emulsion did not affect the creaming index, probably because these emulsions (0.5CP_0.3PR and 0.5CP_0.6PR) were less flocculated than 0.2CP_0.3PR and 0.2CP_0.6PR and also presented higher viscosities. With regard to the OBF emulsions, the droplet migration was diminished when adding proteins. For instance, 1.0OBF_0.3PR and 2.4OBF_0.3PR showed about 40 and 90% less creaming than 1.0OBF and 2.4OBF, respectively. 

Overall, larger quantities of OBF are needed to match the characteristics (e.g., apparent viscosity and creaming index) observed in emulsions produced with pure pectins in equivalent amounts of this polysaccharide. This is interesting since OBF showed a relatively high antioxidant capacity and polyphenol content; those larger amounts of OBF are desirable in food products that are prone to oxidation. 

### 3.3. Artificial Network Modeling

Table 3 shows the results of the two-way ANOVA, taking OBF and protein content as factors. As can be seen, both factors had a significant effect on the characteristics of the emulsion (*p* < 0.05) and, therefore, could be considered input variables for the ANNs.

ANNs with three (input, hidden, and output) layers were developed by using back propagation algorithms and the experimental data in order to predict the characteristics (apparent viscosity; Z potential; percentiles d10, d50, and d90 of the droplet size distribution; percentage of flocculation; and creaming index) of the emulsions containing protein and OBF.

To determine the best ANN structure, several iterations with different numbers of neurons in the hidden layer were carried out (between 2 and 5). Iteratively varying the number of neurons led to the identification of the optimum number of neurons in the hidden layer. From the development of the neuronal networks, it was observed that the number of neurons in the hidden layer depended on the emulsion characteristic evaluated; therefore, three neurons in the hidden layer were applied in all the ANNs developed, except in those corresponding to viscosity, Z potential, and d50, which required four of them. As transfer functions, the hyperbolic tangent sigmoid transfer function “tansig” was used in the hidden layer, and the linear transfer function “purelin” was used for the output layer.

**Figure 5:**
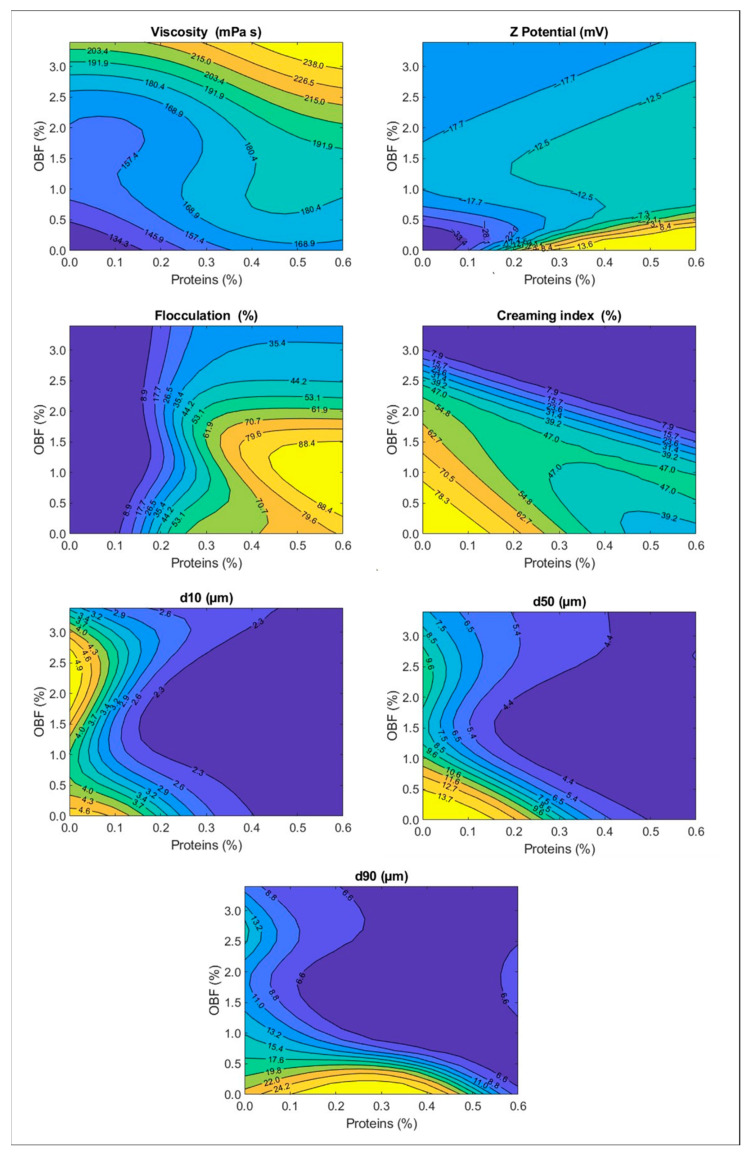
Contour plot of each emulsion characteristic predicted by ANNs at different levels on a protein-content–OBF content plane.

The simulation obtained with the ANNs can be observed in the contour plots shown in Figure 5 for each emulsion characteristic (apparent viscosity; Z potential; percentage of flocculation; creaming index; and percentiles d10, d50, and d90 of the droplet size distribution). This figure depicted the isolines of each emulsion characteristic (dependent variables) at different levels on a protein-content–OBF content plane (independent variables).

In these plots, it can be observed that, for the viscosity, there is a reciprocal effect of protein and OBF content; increasing both resulted in higher viscosities for the emulsions. The Z potential absolute value, on the other hand, had a more complex trend. It decreased gradually when increasing the protein content in the whole range studied. Without proteins or OBF, the oil droplets adsorb OH^−^, and the addition of proteins which are positively charged brings the Z potential value closer to zero. With a protein concentration of 0%, this parameter decreased rapidly when increasing the OBF content from 0 to 1% (its absolute value decreased from about 33 to 18), and thereafter it remained the same. The OBF contains pectins, which are negatively charged polysaccharides, but when they are adsorbed, replacing the OH^−^ anions, they are not as efficient in increasing the negative charge, probably because they are large macromolecules with a lower charge concentration. However, at higher protein content levels (e.g., 0.5%), the Z potential absolute value gradually increased with higher OBF concentrations; thus, the OBF negative charge takes this value beyond zero. The flocculation plot indicates clearly that increasing the protein concentration resulted in a higher flocculation percentage. Moreover, without proteins, the emulsions showed no flocculation in the whole range of OBF content studied. At high protein concentrations (e.g., 0.6%) the flocculation decreased when augmenting the OBF content. This could be due to an increase in steric repulsions because of the thicker interfacial layer when adding OBF, an increase in electric repulsions because of higher Z potential values, and a decrease in the droplets’ mobility because of higher viscosities. Regarding the creaming index, this parameter decreased when increasing both protein and OBF contents. This is related to the higher viscosities observed in emulsions produced with higher content of these materials. Finally, the percentiles of the particle size distribution (d10, d50, and d90) decreased with higher contents of both the proteins and OBF. The effect seemed to be more noticeable for the proteins. For instance, when increasing the protein concentration from 0 to 0.5%, the d50 decreased from about 14 to 4 μm. However, when increasing the OBF concentration from 0.5 to 3%, the d50 decreased from 14 to 9 μm. This suggests that proteins are more easily adsorbed.

On the other hand, Supplementary Figure S2 depicts the simulated results represented versus the experimental data, along with the prediction bounds of the regression (95% of confidence level). This comparison was carried out in order to evaluate the goodness of the model. All the outputs showed a good correlation between the calculated and the experimental results. This could be inferred from the low MRE values (average 2.1 ± 1.1%), and the correlation coefficient values (r^2^), which were close to 1 in all cases (average 0.997 ± 0.005). Moreover, the slope of the straight lines was close to 1, and the y-intercept was close to 0, indicative of low bias and lack of systematic errors. On the other hand, in Supplementary Figure S3A, the residuals are represented in histograms, where it can be seen that they present a normal distribution (*p* > 0.05). Supplementary Figure S3B shows the representation of the residuals vs. the calculated data, and, generally, the residuals were scattered randomly about zero. All the points were in the range of +3 and −3, with the exception of a possible outsider for viscosity (about 5). 

#### Optimization

The optimum values of OBF and protein concentration obtained with the ANNs were 3.16 and 0.14% *w*/*w*, respectively. To evaluate the optimization, an additional emulsion containing those concentrations of OBF and proteins was prepared and analyzed. The oil content was maintained at 6% *w*/*w*, and the maltodextrin content was adjusted at 30.7% *w*/*w* to keep a dry-matter content of 40% *w*/*w*. The experimental results obtained for the characteristics of the optimum emulsion are shown in Table 4, together with the characteristics predicted by the ANNs and the prediction bounds (95%). As can be seen, a satisfactory agreement between experimental and predicted values was observed. None of the emulsion characteristics showed significant differences (*p* > 0.05) between the predicted and the experimental results except for d10, d50, and flocculation, and even for these characteristics, the differences could be considered small (*p* > 0.01).

**Figure 6:**
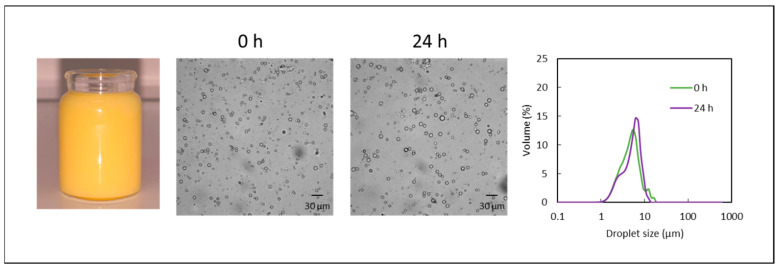
Photograph, micrographs, and droplet size distribution obtained for the optimum emulsion immediately after its preparation (0 h) and after 24 h.

The emulsion prepared with the optimum concentrations of OBF and proteins was orange/yellow and highly stable, with relatively small droplets and few floccules (Figure 6) and with no significant increases (*p* > 0.05) in the d10, d50, and d90 percentiles after 24 h (e.g., d50 at 24 h 5.16 ± 0.81 μm). 

## 4. Conclusions

Overall, it was possible to stabilize emulsions with an orange by-product rich in pectins. By comparing the OBF with the purified pectins, it could be concluded that OBF presented some advantages, such as a higher antioxidant capacity and the presence of polyphenols. Not all the pectins present in OBF seemed to be solubilized in the continuous phase of the emulsions; therefore, higher amounts of this material are needed to increase its viscosity or to improve the stability against creaming when compared with purified pectins. The OBF was able to produce emulsions that were highly stable against coalescence, flocculation (with 2.4 and 3.4% *w*/*w*), and creaming (3.4% *w*/*w*). In combination with proteins, the emulsions prepared with OBF showed smaller droplets, as this is a desirable characteristic for processes such as spray drying, and also decreased the droplet migration (for 1.0OBF and 2.4OBF). However, the flocculation level increased with the addition of proteins, but these floccules did not result in coalescence or greater creaming.

Artificial neural networks have been demonstrated to be useful in the modeling and optimization of the composition of oil-in-water emulsions stabilized with an orange by-product flour rich in pectins and vegetable proteins. The simulation obtained with the trained ANNs allowed us to better understand the effect of the independent variables on each emulsion parameter. Moreover, using the trained ANNs and defining an objective function that describes the desired characteristics of the emulsion, the optimum formulation could be obtained and experimentally validated. Therefore, the proposed methodology can be successfully applied in the formulation of complex food products such as emulsions.

## Figures and Tables

**Table 1:** Composition of the emulsions prepared in g/100 g of emulsion. All the emulsions contained 60% *w*/*w* of water and 6% *w*/*w* of oil with or without proteins (PR), commercial pectins (CP), and an orange by-products flour (OBF).

Batch	Code	CP	OBF	Protein	MD
Control	Control	-	-	-	34.00
PR	0.3PR	-	-	0.30	33.70
	0.6PR	-	-	0.60	33.40
CP	0.2CP	0.20	-	-	33.80
	0.5CP	0.50	-	-	33.50
CP–PR	0.2CP_0.3PR	0.20	-	0.30	33.50
	0.2CP_0.6PR	0.20	-	0.60	33.20
	0.5CP_0.3PR	0.50	-	0.30	33.20
	0.5CP_0.6PR	0.50	-	0.60	32.90
OBF	1.0OBF	-	0.95	-	33.05
	2.4OBF	-	2.38	-	31.62
	3.4OBF	-	3.40	-	30.60
OBF–PR	1.0OBF_0.3PR	-	0.95	0.30	32.75
	1.0OBF_0.6PR	-	0.95	0.60	32.45
	2.4OBF_0.3PR	-	2.38	0.30	31.32
	2.4OBF_0.6PR	-	2.38	0.60	31.02
	3.4OBF_0.3PR	-	3.40	0.30	30.30
	3.4OBF_0.6PR	-	3.40	0.60	30.00

**Table 2:** Composition and antioxidant activity of the orange by-product flour (OBF).

Moisture (g/100 g dm)	6.00 ± 0.49
Lipid (g/100 g dm)	1.12 ± 0.04
Proteins (g/100 g dm)	3.63 ± 0.26
Ashes (g/100 g dm)	3.04 ± 0.22
Total carbohydrates (g/100 g dm)	93.38 ± 1.61
Total fiber (g/100 g dm)	38.18 ± 3.12
Carbohydrate composition (g/100 g dm)	
Uronic acid	16.26 ± 0.22
Arabinose	3.85 ± 0.36
Galactose	2.12 ± 0.19
Rhamnose	0.11 ± 0.01
Glucose	2.73 ± 0.32
Xylose	0.90 ± 0.11
Mannose	0.52 ± 0.03
Fucose	0.10 ± 0.01
Pectins	22.34 ± 1.00
Degree of methylation (%)	41.12 ± 2.11
Total polyphenols content (mg GAE/g dm)	16.20 ± 1.91
Antioxidant activity (CUPRAC) (mg TE/g dm)	25.44 ± 1.24

**Table 3:** Results of the 2-way ANOVA, taking OBF and protein content as factors.

Emulsion Characteristic	Factor	Degrees of Freedom	Sums Square	Means Square	F Value	*p*-Value
Apparent viscosity	OBF	3	33,753	11,251	372.706	***
	Proteins	2	10,047	5024	166.416	***
	OBF:Proteins	6	889	148	4.909	**
	Residuals	24	724	30		
Z potential	OBF	3	1485	494.9	340.4	***
	Proteins	2	2441	1220.7	839.7	***
	OBF:Proteins	6	4018	669.7	460.7	***
	Residuals	24	35	1.5		
d10	OBF	3	1.79	0.597	5.176	***
	Proteins	2	33.40	16.698	144.669	***
	OBF:Proteins	6	4.70	0.784	6.791	***
	Residuals	24	2.77	0.115		
d50	OBF	3	77.3	25.76	329.1	***
	Proteins	2	333.7	166.85	2131.3	***
	OBF:Proteins	6	56.0	9.33	119.1	***
	Residuals	24	1.9	0.08		
d90	OBF	3	836.8	278.93	521.4	***
	Proteins	2	559.7	279.86	523.2	***
	OBF:Proteins	6	496.6	82.77	154.7	***
	Residuals	24	12.8	0.53		
Flocculation	OBF	3	5291	1764	242.4	***
	Proteins	2	27,022	13,511	1856.8	***
	OBF:Proteins	6	4616	769	105.7	***
	Residuals	24	175	7		
Creaming index	OBF	3	23,548	7849	1743.9	***
	Proteins	2	7222	3611	802.3	***
	OBF:Proteins	6	3166	528	117.2	***
	Residuals	24	108	5		

Significance codes: *** *p* < 0.001; ** *p* < 0.01.

**Table 4:** Predicted [prediction bounds (95%)] and experimental values of the optimum emulsion characteristics.

Emulsion Characteristic	Predicted	Experimental
Viscosity (mPa·s)	200 [193, 206]	190 ± 10
Z potential (mV)	−19.3 [−18.1, −20.7]	−18.5 ± 0.7
d10 (μm)	2.97 [2.64, 3.20]	2.22 ± 0.13
d50 (μm)	6.06 [5.70, 6.25]	4.56 ± 0.77
d90 (μm)	7.50 [6.81, 8.31]	7.31 ± 0.65
Flocculation (%)	2.8 [1.6, 3.4]	5.9 ± 0.4
Creaming index (%)	0.6 [−0.7, 1.1]	0.0 ± 0.0

## Data Availability

Data are contained within the article or Supplementary Materials.

## Supplementary Materials

The following supporting information can be downloaded at https://www.mdpi.com/article/10.3390/foods11233750/s1. Figure S1: Photographs of the emulsions containing purified commercial pectins (0.2CP and 0.5CP for 0.2 and 0.5% *w*/*w*, respectively) or an orange by-product flour (1.0OBF, 2.4OBF, and 3.4OBF for 0.95, 2.38, and 3.40% *w*/*w*, respectively) combined with proteins (0.3PR and 0.6PR for 0.3 and 0.6% *w*/*w*, respectively) or without them (0PR). Figure S2: Calculated (calc) vs. experimental (average ± deviation) (exp) emulsions’ characteristics: percentiles of the droplet size distribution (d10, d50, and d90), flocculation, apparent viscosity, Z potential, and creaming index. Figure S3A: Histogram of residuals of the emulsions’ properties: percentiles of the droplet size distribution (d10, d50, and d90), flocculation, apparent viscosity, Z potential, and creaming index. Figure S3B: Residuals vs. calculated values of the emulsions’ properties: percentiles of the droplet size distribution (d10, d50, and d90), flocculation, apparent viscosity, Z potential, and creaming index.
