# Supplementary material for: Artificial Neural Networks to Optimize Oil-in-Water Emulsion Stability with Orange By-Products
Source: Foods. 2022 Nov 22;11(23):3750. doi: 10.3390/foods11233750 (PMC9739075; doi:10.3390/foods11233750)
Supplement: Supplementary file 1 [file foods-11-03750-s001.zip › Figure S1.pdf]

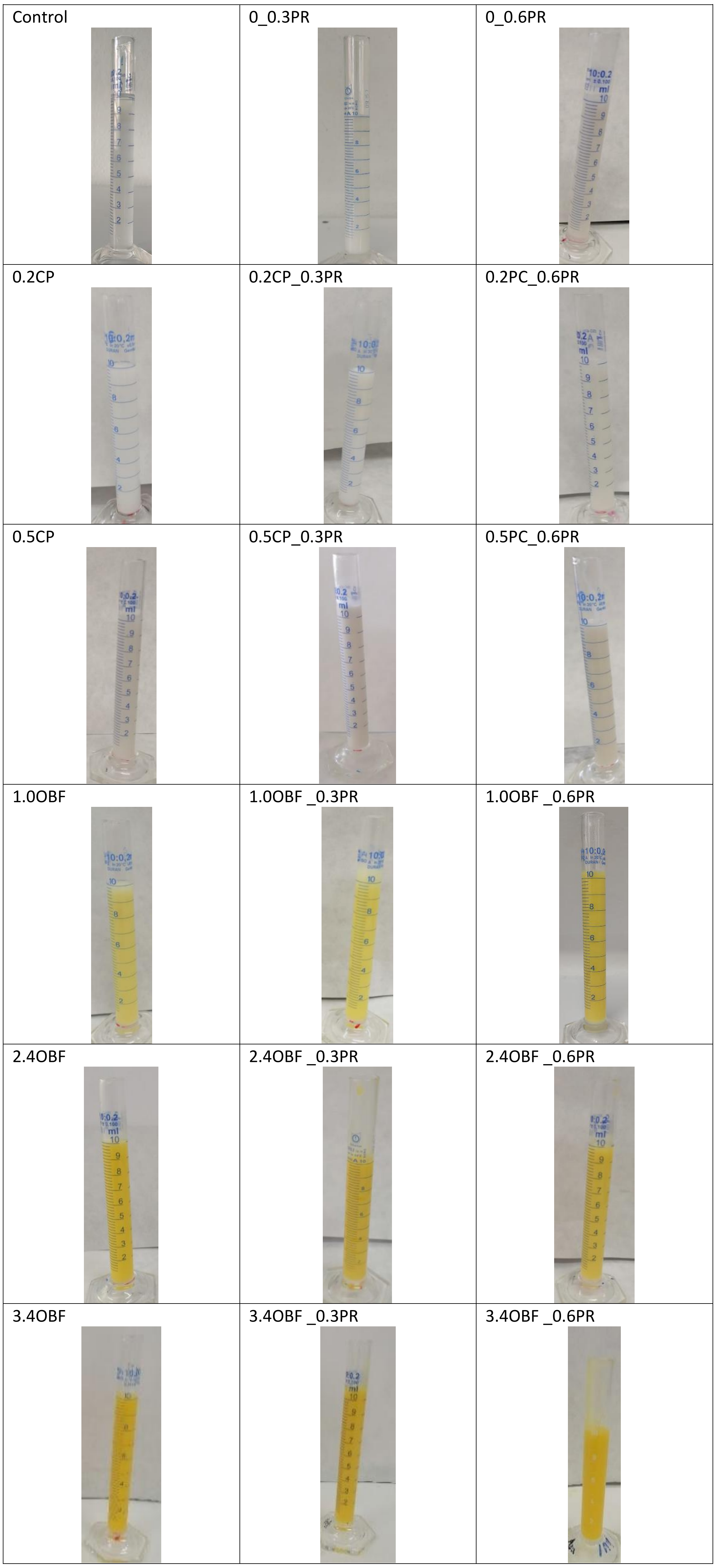

Figure S1: Photographs of the emulsions without any source of pectins or proteins (Control), containing purified commercial pectins (0.2CP and 0.5CP for 0.2 and 0.5 % w/w, respectively) or an orange by-product flour (1.0OBF, 2.4OBF, and 3.4OBF for 0.95, 2.38. and 3.40 % w/w, respectively) combined with proteins (0.3PR and 0.6PR for 0.3 and 0.6 % w/w, respectively) or without them (0PR).
