# Supplementary material for: Artificial Neural Networks to Optimize Oil-in-Water Emulsion Stability with Orange By-Products
Source: Foods. 2022 Nov 22;11(23):3750. doi: 10.3390/foods11233750 (PMC9739075; doi:10.3390/foods11233750)
Supplement: Supplementary file 1 [file foods-11-03750-s001.zip › Figure S2.pptx]

## Slide 1
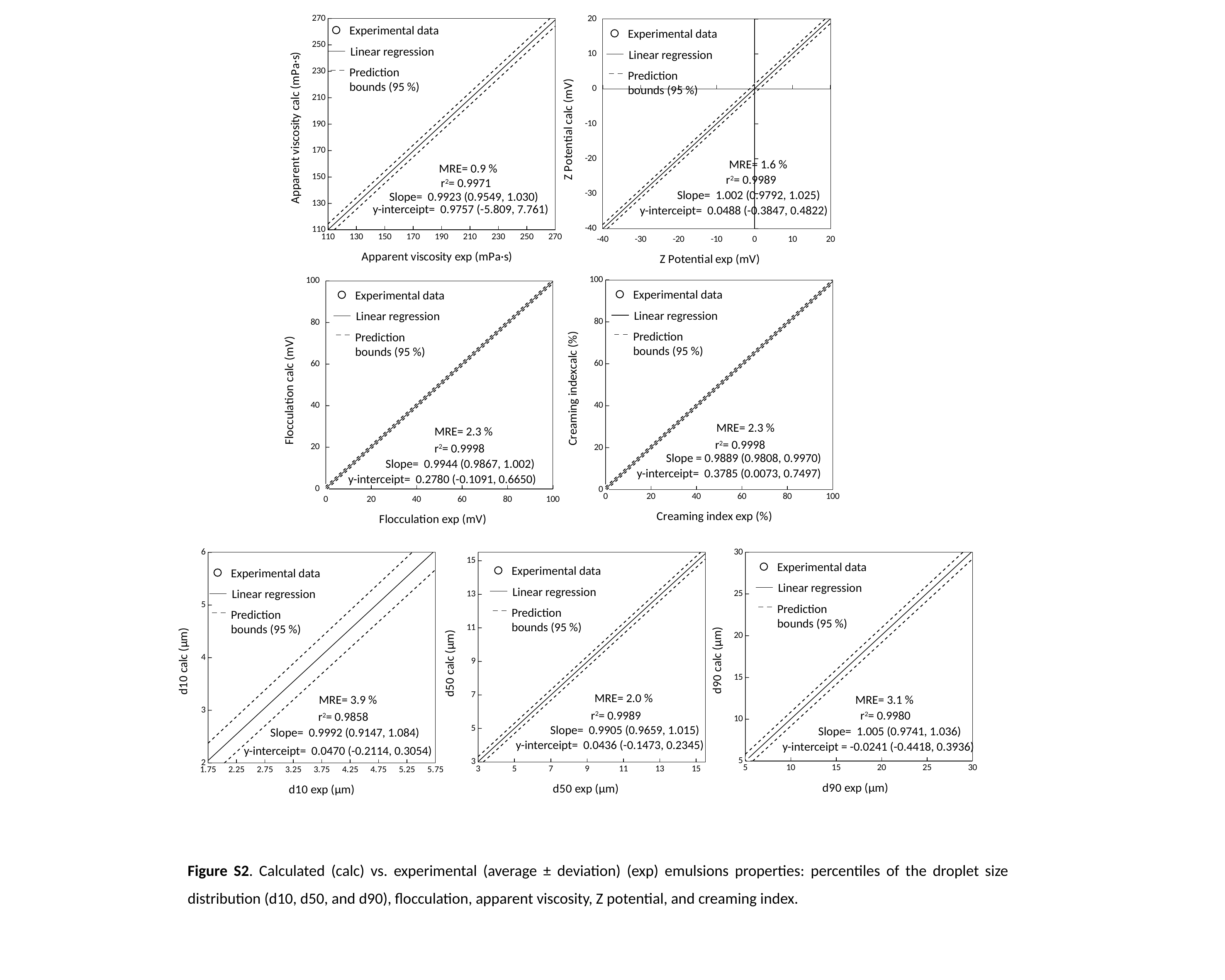

### Chart
| Category | vis | ci i | ci s |
|---|---|---|---|Experimental data
Linear regression
Prediction bounds (95 %)
MRE= 0.9 %
r2= 0.9971
Slope= 0.9923 (0.9549, 1.030)
y-interceipt= 0.9757 (-5.809, 7.761)
### Chart
| Category | potz | ci i | ci s |
|---|---|---|---|Experimental data
Linear regression
Prediction bounds (95 %)
MRE= 1.6 %
r2= 0.9989
Slope= 1.002 (0.9792, 1.025)
y-interceipt= 0.0488 (-0.3847, 0.4822)
### Chart
| Category | ci | ci i | ci s |
|---|---|---|---|
### Chart
| Category | potz | ci i | ci s |
|---|---|---|---|Experimental data
Linear regression
Prediction bounds (95 %)
MRE= 2.3 %
r2= 0.9998
Slope= 0.9944 (0.9867, 1.002)
y-interceipt= 0.2780 (-0.1091, 0.6650)
Experimental data
Linear regression
Prediction bounds (95 %)
MRE= 2.3 %
r2= 0.9998
Slope = 0.9889 (0.9808, 0.9970)
y-interceipt= 0.3785 (0.0073, 0.7497)
### Chart
| Category | d50 | ci i | ci s |
|---|---|---|---|
### Chart
| Category | d50 | ci i | ci s |
|---|---|---|---|
### Chart
| Category | d10 | ci i | ci s |
|---|---|---|---|Experimental data
Linear regression
Prediction bounds (95 %)
Experimental data
Linear regression
Prediction bounds (95 %)
Experimental data
Linear regression
Prediction bounds (95 %)
MRE= 2.0 %
MRE= 3.9 %
MRE= 3.1 %
r2= 0.9989
r2= 0.9980
r2= 0.9858
Slope= 0.9905 (0.9659, 1.015)
Slope= 1.005 (0.9741, 1.036)
Slope= 0.9992 (0.9147, 1.084)
y-interceipt= 0.0436 (-0.1473, 0.2345)
y-interceipt = -0.0241 (-0.4418, 0.3936)
y-interceipt= 0.0470 (-0.2114, 0.3054)
Figure S2. Calculated (calc) vs. experimental (average ± deviation) (exp) emulsions properties: percentiles of the droplet size distribution (d10, d50, and d90), flocculation, apparent viscosity, Z potential, and creaming index.
