# Supplementary material for: Artificial Neural Networks to Optimize Oil-in-Water Emulsion Stability with Orange By-Products
Source: Foods. 2022 Nov 22;11(23):3750. doi: 10.3390/foods11233750 (PMC9739075; doi:10.3390/foods11233750)
Supplement: Supplementary file 1 [file foods-11-03750-s001.zip › Figure S3.pptx]

## Slide 1
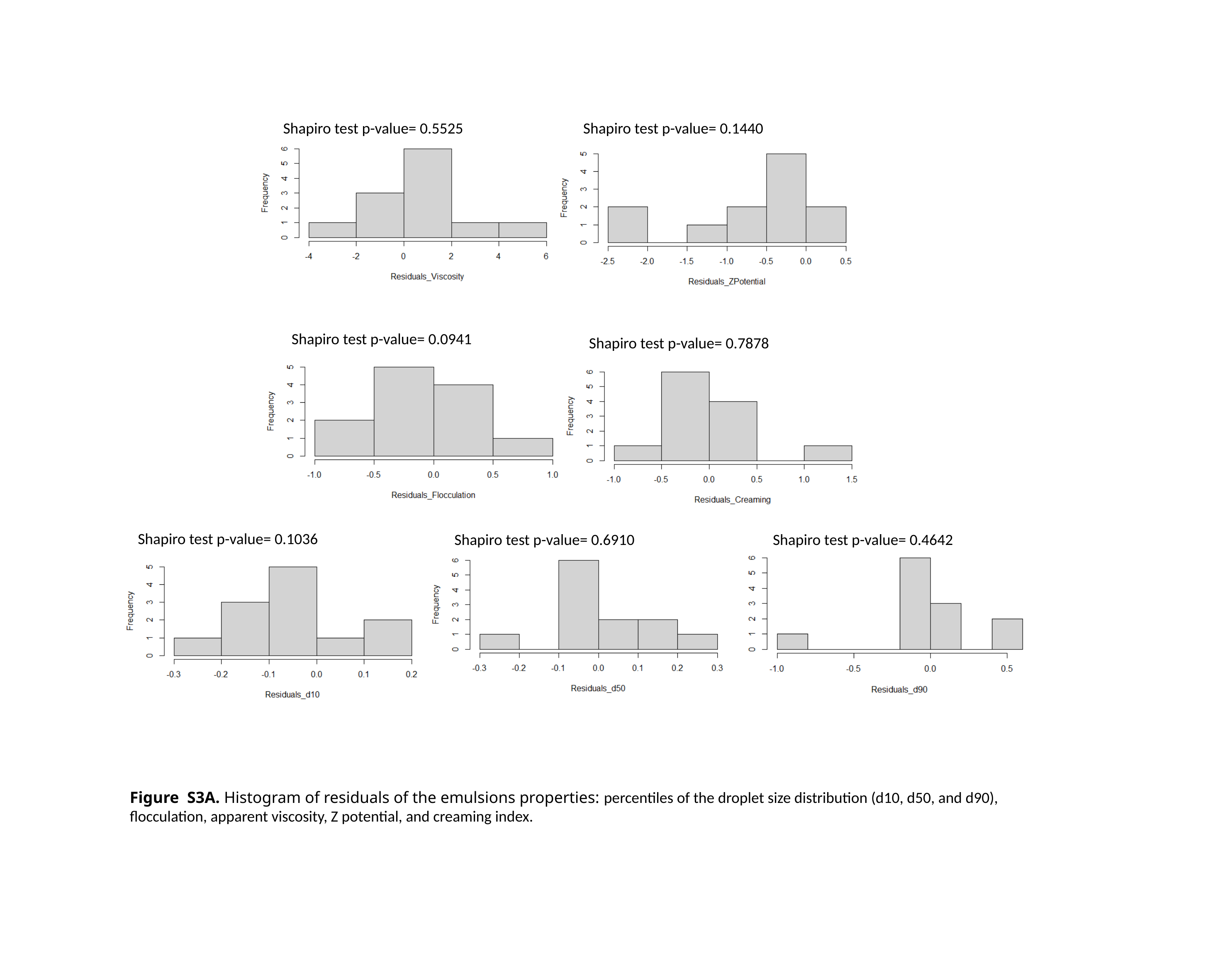

Shapiro test p-value= 0.5525
Shapiro test p-value= 0.1440
Shapiro test p-value= 0.0941
Shapiro test p-value= 0.7878
Shapiro test p-value= 0.1036
Shapiro test p-value= 0.6910
Shapiro test p-value= 0.4642
Figure S3A. Histogram of residuals of the emulsions properties: percentiles of the droplet size distribution (d10, d50, and d90), flocculation, apparent viscosity, Z potential, and creaming index.

## Slide 2
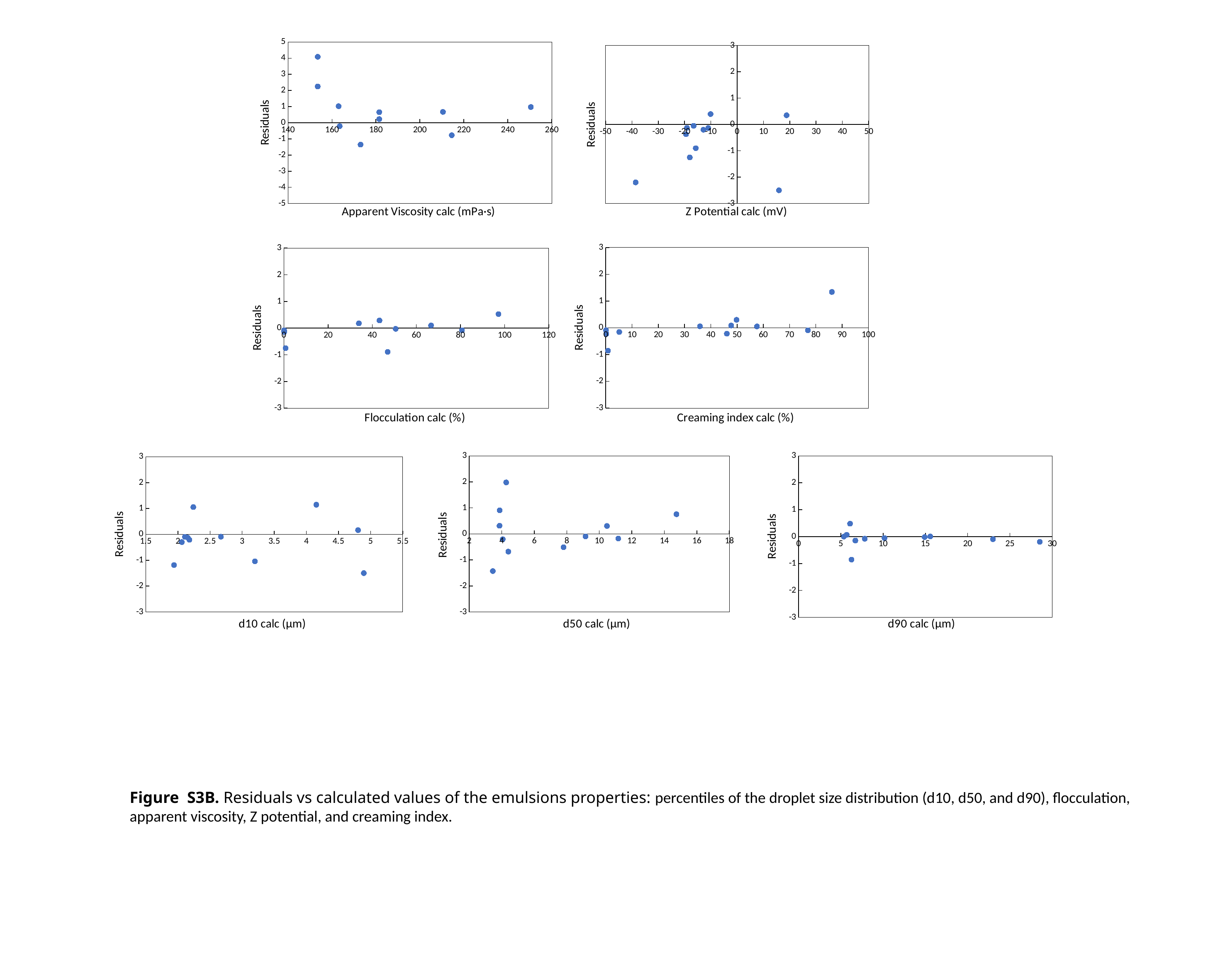

### Chart
| Category | |
|---|---|
### Chart
| Category | |
|---|---|
### Chart
| Category | |
|---|---|
### Chart
| Category | |
|---|---|
### Chart
| Category | |
|---|---|
### Chart
| Category | |
|---|---|
### Chart
| Category | |
|---|---|Figure S3B. Residuals vs calculated values of the emulsions properties: percentiles of the droplet size distribution (d10, d50, and d90), flocculation, apparent viscosity, Z potential, and creaming index.
